# 3D printable biomimetic rod with superior buckling resistance designed by machine learning

**DOI:** 10.1038/s41598-020-77935-w

**Published:** 2020-11-26

**Authors:** Adithya Challapalli, Guoqiang Li

**Affiliations:** Department of Mechanical & Industrial Engineering, Louisiana State University, Baton Rouge, LA 70803 USA

**Keywords:** Engineering, Mathematics and computing

## Abstract

Our mother nature has been providing human beings with numerous resources to inspire from, in building a finer life. Particularly in structural design, plenteous notions are being drawn from nature in enhancing the structural capacity as well as the appearance of the structures. Here plant stems, roots and various other structures available in nature that exhibit better buckling resistance are mimicked and modeled by finite element analysis to create a training database. The finite element analysis is validated by uniaxial compression to buckling of 3D printed biomimetic rods using a polymeric ink. After feature identification, forward design and data filtering are conducted by machine learning to optimize the biomimetic rods. The results show that the machine learning designed rods have 150% better buckling resistance than all the rods in the training database, i.e., better than the nature’s counterparts. It is expected that this study opens up a new opportunity to design engineering rods or columns with superior buckling resistance such as in bridges, buildings, and truss structures.

## Introduction

Our mother nature took millions of years to form, create and develop itself into what it is today. Through evolution, nature modifies its elements to sustain, develop and flourish around their environments. Various species have evolved and modified slowly over thousands and millions of years. The plant kingdom is one of the very old and major species of nature that controls and supports many other species and systems on Earth. With slow but conscientious evolution, plant species educate humankind towards numerous scientific and research breadths. By studying nature and the evolution of plants, humans were able to understand Earth and ecosystem better, develop cultivation, form civilizations and advance in technology from the stone-age. Humans have used and mimicked nature to design and improve their tools for a better living.

Many studies have been focused on the biology of plants to understand and mimic various aspects in developing new technologies that surpass conventional manmade objects. A few of them include the self-cleaning mechanism of aquatic plants like the lotus. The super hydrophobicity of the lotus flowers has been mimicked to design facade paints, tiles, self-cleaning glasses and surfaces where hydro-degradation occurs^1,2^. Biohybrid systems such as microfluidic devices have been developed by inspiration from the fluid transportation of plants with the light energy stored in them through photosynthesis^3,4^. The transportation of fluids and waters in the plants is also taken as inspiration in textile fabrications. To provide better comfortability in humid conditions, moisture management fabrics can provide water transportation formed as sweat to the outer surface of the fabric. Plant structures are mimicked to design these fabrics more effectively than conventional fabrics^5^. Velcro’s are designed by George de Mestral inspired by the hooks of plant burrs^6^. Pummelo is taken as an inspiration to develop structures with excellent damping properties^7^. This fruit has a thick layer of skin around its pulp that is in the form of porous layers. The skin prevents the fruit from impact damage when it hits the ground. Metallic foam structures with low weight and good damping properties were developed by studying and mimicking this feature of the fruit. The biomimicry of plants is also applied in architectural design. The eye-catching shapes of flowers such as lily and plant cells were mimicked to design furniture^8^. Bamboo which is considered as a strong composite material is studied for structures with better structural capacities. The helical reinforcement of bamboo fibers is investigated for applications in developing engineering composite materials^9^. The tendril structure from plants has been mimicked to develop polymeric artificial muscles by twist insertion in precursor fibers^10,11^. Scanning Electron Microscopy helped in observing the microscale surfaces of hydrophobic plants, which lead to the understanding of the self-cleaning behavior of those plants^12^. Additive manufacturing and various design tools help in the fabrication of very complicated structures, which were not possible by other conventional manufacturing methods.

Buckling is a major failure mode for slender columns or rods subjected to axial compression^13^. This type of failure mode should be avoided in structural design so that the load-carrying capacity of materials can be fully utilized. Traditionally, columns or rods are optimized in terms of its geometrical shape such as drum-shaped rods have higher buckling load than uniform cylinders^14^. Because the materials around the rod axis do not provide much bending resistance, hollow or porous rods usually have higher buckling resistance than solid rods with the same amount of materials^15,16^. As discussed above, plant stem and root usually have porous structures. Therefore, mimicking their porous structures may be a way of developing manmade columns or rods with superior buckling resistance.

**Figure 1:**
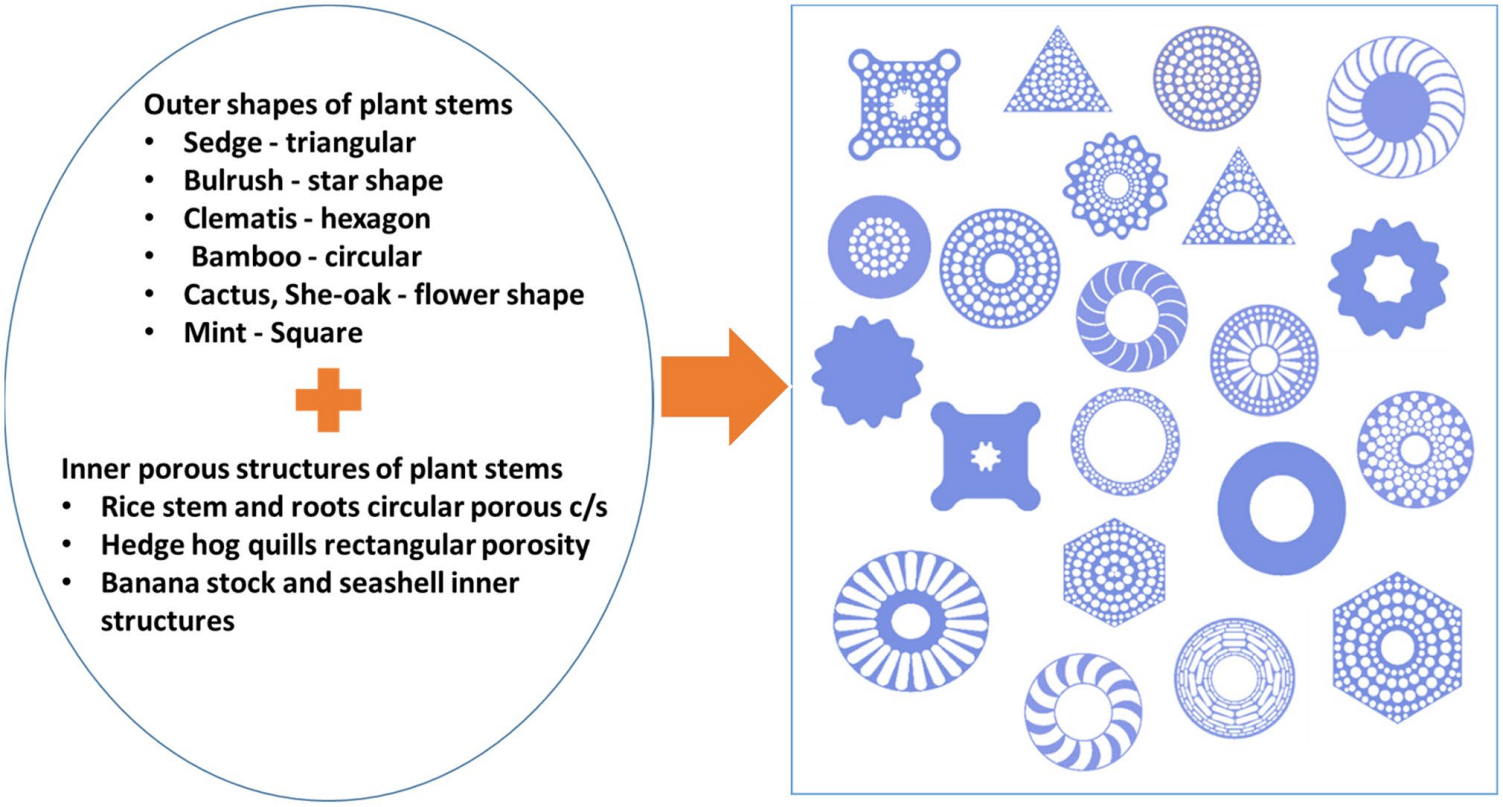
Schematic of biomimetic structures learned from the nature. Biological structures with various external shapes and internal porous structures (on the left), and the cross-sectional view of biomimetic rods through a combination of external shape and internal microstructure of biological structures (on the right).

Porous structures are popular not only in plants but also in seashell structures, animal quills and honeycombs; see a schematic on the left-hand side in Fig. 1. These natural structures can be idealized as biomimetic structures shown on the right-hand side in Fig. 1. Based on our understanding of buckling resistance, these porous structures are good candidates to develop biomimetic rods with superior buckling resistance.

The buckling mode in the form of lateral deflection is a common type of failure observed in long rods under compression. Inspired by the biological structures, the objective of this study is to design columns or rods with superior buckling resistance. Each biological structure is then digitalized to determine its features or fingerprints, which form a training database. This database is modeled by machine learning to establish the correlation between the features or fingerprints and the buckling load. Data filtering is then conducted to optimize the biological structures with a goal of learning from nature but better than nature. To this end, first, the biological structures are idealized and modeled using finite element analysis (FEA) to obtain their buckling loads. Some typical biomimetic rods are 3D printed and tested to validate the FEA predictions. It is noted that Euler buckling equation can be used to determine global buckling load directly for solid rods under ideal boundary conditions such as simply supported or fixed boundary conditions. For the biomimetic rods in this study, the complex geometrical shape makes calculation of the bending stiffness extremely difficult. Therefore, Euler buckling equation cannot be used directly. This is why we used FEA to provide the training database and used machine learning to speed up the discovery of optimized biomimetic rods.

## Results

### Buckling load analysis of the biomimetic rods

**Figure 2:**
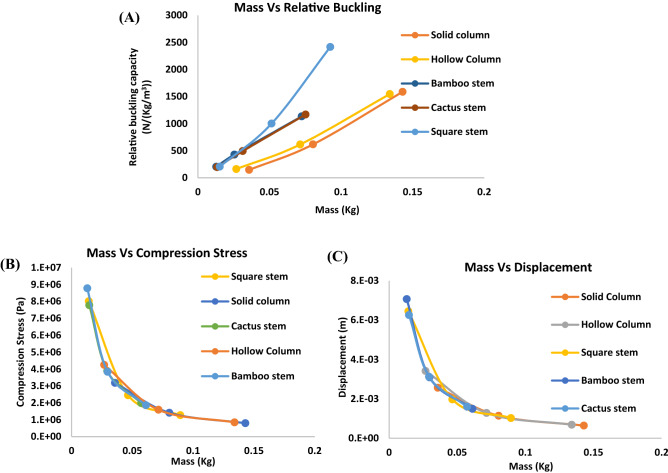
Simulation results from ANSYS^17^ conducted on control solid rods, hollow rods, and biomimetic rods. Mass vs (**A**) normalized buckling capacity, (**B**) compression stress, and (**C**) axial displacement, respectively.

The mass vs. normalized buckling capacity (since the overall volume is constant for all the designs), compressive stress, and axial displacement during stress and buckling analysis for several representative biomimetic rods are shown in Fig. 2. More stress analysis and buckling analysis for additional biomimetic rods are presented in Fig. S1 in Supplementary Information (SI).

It is seen from Fig. 2A that the normalized buckling capacities of the biomimetic rods are more than twice that of solid and hollow rods with the same mass. Therefore, the biomimetic rods show a considerable improvement in terms of buckling resistance. However, our goal is to mimic nature but exceed the nature counterparts. Therefore, we will use machine learning to further improve the biomimetic rods design, which will be discussed in the following sections.

However, one question that needs to be answered before conducting machine learning is that the rods did not fail before buckling occurred. This was answered through stress analysis. From Fig. 2B, the peak compressive stress in the biomimetic rods is similar to that in the solid rod when they have the same mass. Of course, biomimetic rods can be very lightweight, and for these very lightweight rods, their peak compressive stress is high. However, as compared to the compressive strength of the polymer in Table S2, even the stress in the lightest rod is far below the polymer strength. Therefore, compression failure is avoided.

As for the axial displacement obtained by stress analysis, a similar tendency to compressive stress is seen, i.e., with the same mass, biomimetic rods have similar axial displacement to that in control solid and hollow rods; see Fig. 2C.

From Fig. 2, both the external shape and internal porous structure of the biomimetic rods have a significant impact on the buckling load. It can be seen from Fig. 2A that cactus and square-shaped stems are slightly better as compared to the bamboo stem which has a circular external shape. It is worthwhile to note that all these rods mimic basic stem cross-sections which consist of xylem (hollow cylinders) other than roots which are porous all the way. The inner radius of these rods can be varied to increase the buckling resistance of the structures. As for the effect of inner pores distribution, it is observed from Fig. S1 and Table S4 that designs with continuous porous inner structure as in the bamboo stem perform better than designs in the quill inner structure of a hedgehog, which has scattered pores. The cross-sections mimicking roots, animal quills, seashell inner and outer constructions were outperformed by stem like hollow cylinders in terms of buckling capacity and compression strength; see Fig. S1. Therefore, by altering and combining various structural features of these bio mimic structures, much better designs with higher buckling strengths can be achieved.

### Experimental validation

**Figure 3:**
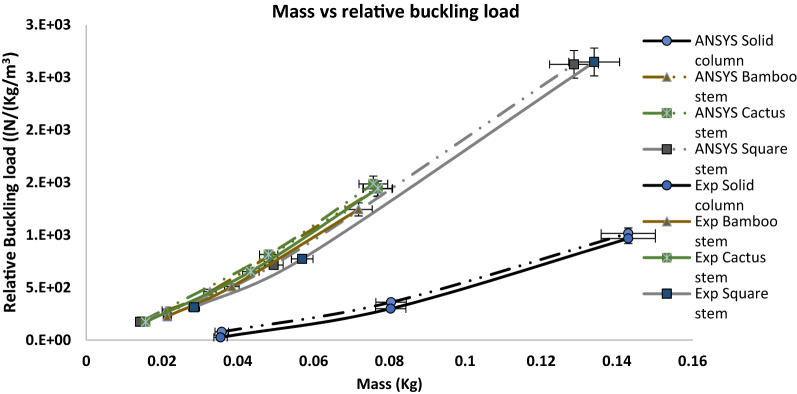
Experimental validation for buckling capacity conducted on biomimetic rods. Four groups of biomimetic rods were used for validation: Bamboo stem, Cactus square, Square stem, and Solid.

Comparisons between the 3D printed biomimetic rods and FEA modeled rods in terms of normalized buckling loads are depicted in Fig. 3. It is seen that the experimental results agree with the simulation results. A slight difference is found between the experimental and simulation, which is primarily due to the compromise in the 3D printing due to low printing resolution^18^. It should be noted that the boundary conditions for the rods in the simulations were adjusted to suit the experimental conditions. Table S3 gives the comparison of the buckled shape between the FEA modeling and experimental testing for several typical biomimetic rods, again, with a good agreement. Therefore, the FEA is validated. The validated FEA will be utilized to calculate the buckling load of the optimized biomimetic rods later.

From this initial investigation, it was observed that directly mimicking these structures in obtaining optimum designs has a couple of limitations. Firstly, it is too strenuous and not ideal to manually design, analyze and compare all the possible rods. Secondly, although an optimum set of structures can be deduced from the designs, it is believed that much better designs with higher structural capacities exist and it is impossible to explore all the possible structures or the structure design space by combining these biomimetic designs. Therefore, machine learning was used to help identify the optimal rods.

### Correlation between buckling load and fingerprints though forward design

**Figure 4:**
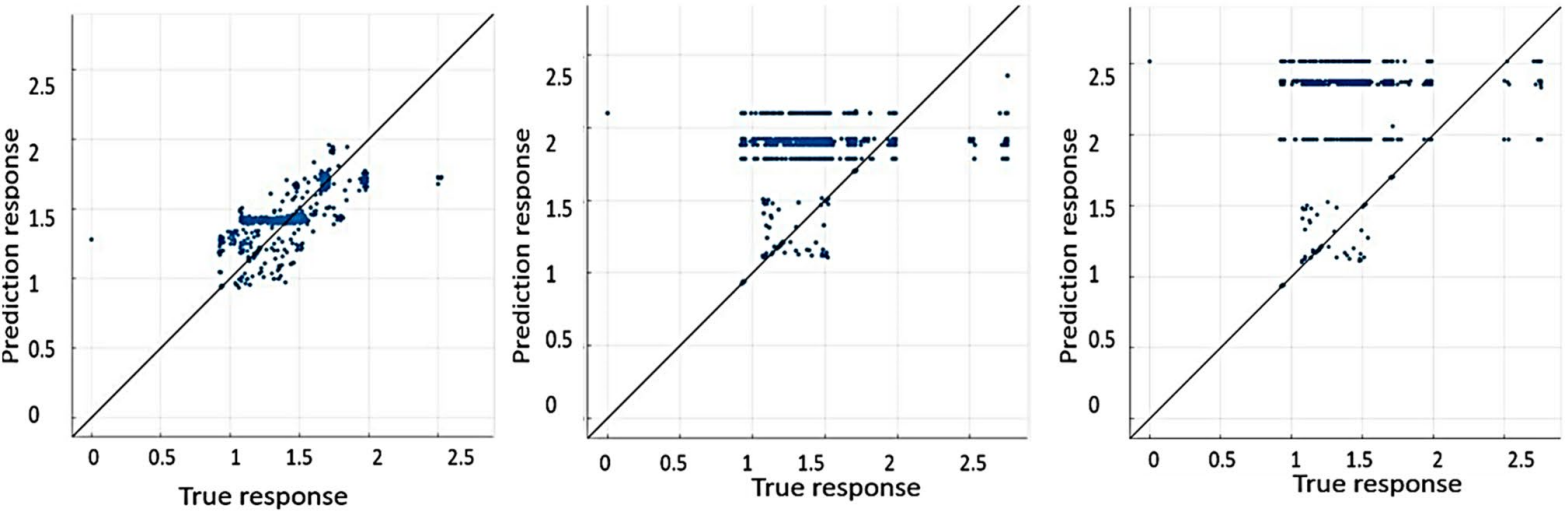
Comparison of predictions vs. true responses. From left to right, they are Ensemble tree, Support vector machines, and Gaussian process regression for buckling capacities of the training dataset.

Figure 4 shows the predictions by machine learning vs. true response or observations by FEA. With the line being predictions and the dots being the observations, the closer the observations to the prediction line, the better the model is. From Fig. 4, it is clear that the ensemble tree model gives the best prediction.

In the ensemble tree approach, the data set is randomly divided into different subsets by the algorithms within itself for predictions. Though precise values for predictions may not be achieved by ensemble tree as it is based on the mean of predictions from the subsets, this model is very advantageous in handling sophisticated data. Since the fingerprints of the current data type contain very large vectors, ensemble tree seems to be a fitful model for regression with this particular data type. Once the model was ready it was exported to the MATLAB^19^ workspace for testing. The test data was made into a table matching the labels as the training data and imported into the workspace. The ‘yfit’ functions were used to predict the buckling strengths of the test data. The error percentage was calculated to be less than 10% for the majority of the test data, except for a few dummy points where it exceeded 10%. This margin of errors has also been used previously by others^20–22^. It is noted that, although the margin of errors have been used, a better comparison of the root mean squared error (RMSE) between the training and testing data would be useful when commenting on any overfitting, which can be an issue with ensemble trees. This will be considered in our future studies. Hence this model can be used to predict the buckling strength of any column containing the features of the trained models instantly.

### Optimization of biomimetic rods

**Figure 5:**
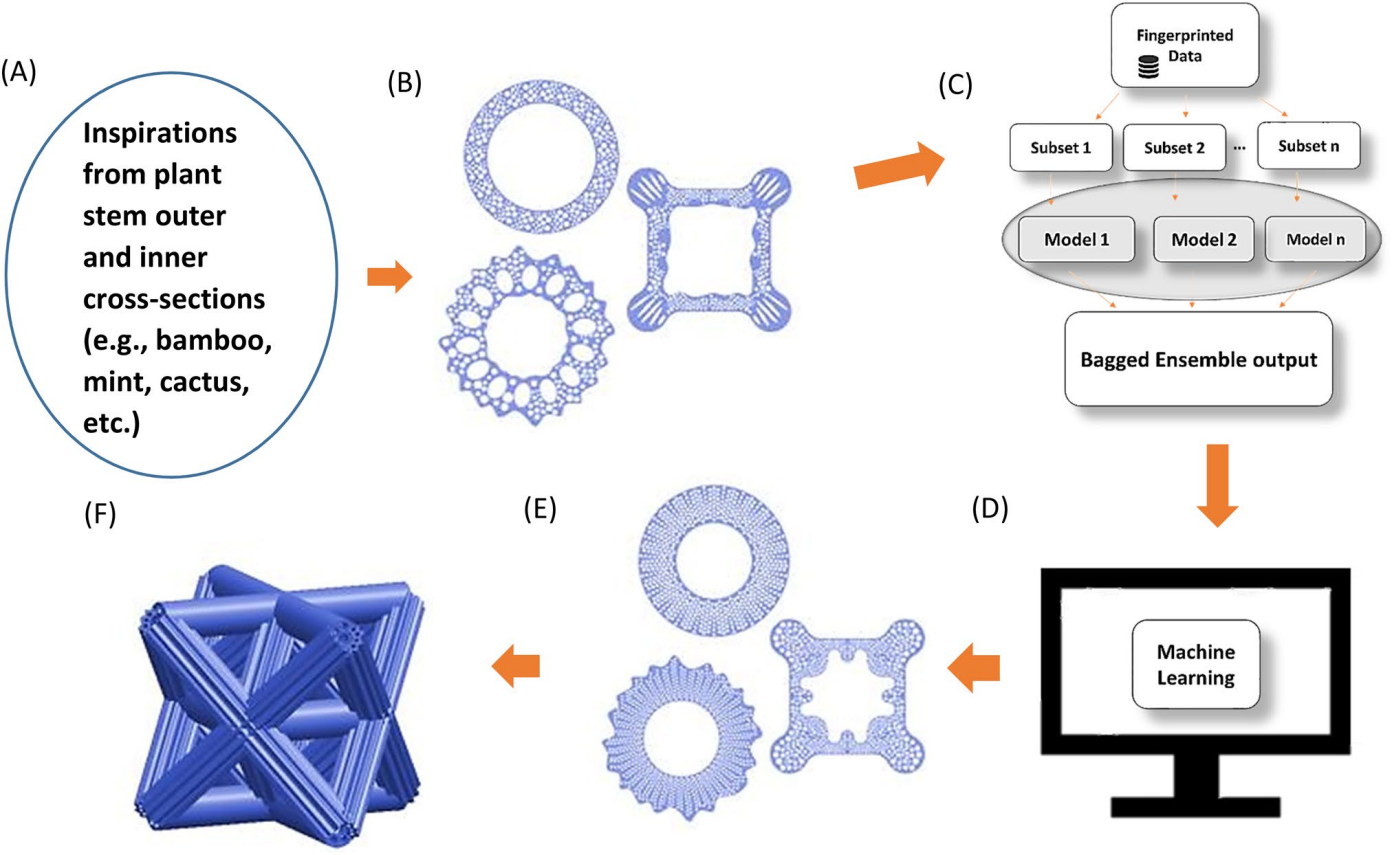
Machine learning assisted design of optimal biomimetic rods and lattice unit cell. (**A**) Biomimetic inspirations, (**B**) initial designs, (**C**) forward machine learning predictions through bagged ensemble tree, (**D**) data filtering to find optimal biomimetic structures, (**E**) CAD designs of single optimum rods (top view), and (**F**) potential application (3D printed lattice structure containing optimal bio-inspired rods).

Figure 5 shows the machine learning framework, including potential applications in biomimetic lattice structures. After the optimization, a total of 160 new rods were created, which are all better than the 1500 rods in the initial training database. Table S5 summarizes the 160 new designs.

**Figure 6:**
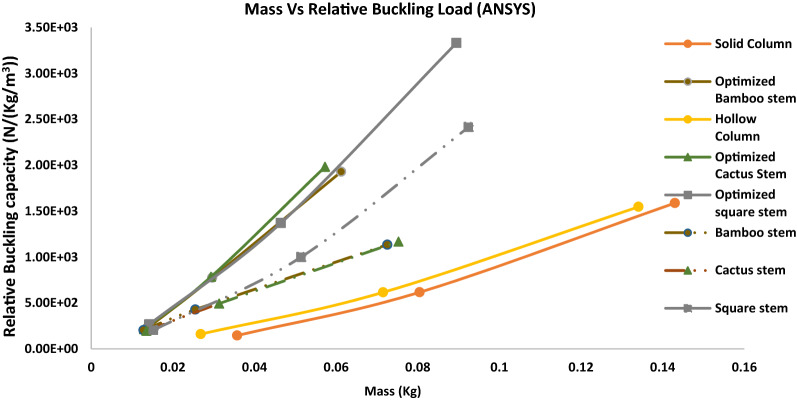
Simulation results from ANSYS conducted on various optimized rods through machine learning, as well as representative rods from the initial 1500 training rods.

In order to determine the buckling load of the newly optimized rods, the validated FEA was used again. Uniaxial compression modeling conducted on these new designs used the same boundary conditions as the modeling conducted on the biomimetic rods. The results are reported in Fig. 6. It is seen from Fig. 6 that the optimized rods inspired from the biomimetic rods through optimization exhibit a buckling strength nearly double that of the biomimetic rods in the initial training dataset. Figure S2 shows the stress distribution for several optimized rods.

## Conclusions

Biomimetic rods were created and optimized through machine learning, with superior buckling properties. Through the combination of the external shape and internal porous structure of several biological systems, a total of 1500 biomimetic rods were created and modeled using ANSYS for their buckling load and stress. Some typical biomimetic rods were then 3D printed and tested for validation of the finite element analysis (FEA). The bagged ensemble tree algorithm was used for forward design to establish the correlation between the buckling load and fingerprints of the 1500 biomimetic rods in the training dataset. Data filtering through MATLAB coding, EXCEL functions and machine learning was then implemented to optimize these biomimetic designs and obtained 160 new rods. It was observed that without causing much additional stresses, the optimized biomimetic rods possess buckling loads several times higher than those of the classical solid or hollow cylinders widely used in engineering structures. This study opens up an opportunity to design lighter weight rods with much improved buckling resistance.

## Methods

### Selection of biological counterparts and creation of biomimetic rods

In this study, biomimetic rods with a combination of the external shape (upper left in Fig. 1) and internal microstructure (bottom left in Fig. 1) in biological counterparts are created as shown on the right-hand side in Fig. 1. From the schematics on the right-hand side in Fig. 1, we created 21 basic biomimetic rods. In particular, the external stem shapes of the rice plant, bamboo, cactus, square (mint, cup plant), bulrush, papyrus and she-oak plants, and the internal porous structures of roots, hedgehog quills, seashells and honeycomb were combined to create the biomimetic rods. However, to conduct machine learning, 21 rods are not enough. In this study, a total of 1500 rods were created for modeling. These additional rods were created based on the 21 basic rods, by making modifications such as changing the shape of pores, pore size distributions, pore locations in the rods, etc. The number of additional rods created for each group can be found in Table S1 in the Supplementary Information (SI).

In Table S1, the biomimetic rods were arranged into 7 groups based on their external shape. To keep consistency, each column or rod had the same height of 10 cm and the same overall volume (volume of the solid material + volume of the pores). We used a circular rod with a diameter of 1 cm to determine the volume, which was 7.85cm^3^. For comparison purpose, both solid cylinder and hollow cylinder (1 cm outer diameter and 0.5 cm inner diameter) were used as control rods. In addition to the 1 cm outer diameter for both solid and hollow rods, two additional outer diameters of 1.5 cm and 2.0 cm were also investigated, leading to a total of 6 control rods.

### Buckling Load analysis of the biomimetic rods

For the biomimetic rods created in Table S1, their buckling load is largely unknown. Therefore, finite element analysis (FEA) was used to determine the buckling load for each biomimetic rod. ANSYS workbench was used to conduct stress and buckling analysis. 3D printable Polylactic Acid (PLA) manufactured by Hatchbox was used as the common material for all the designs. The mechanical properties under uniaxial compression of PLA were evaluated using a Q-TEST 150 machine. For this purpose, ASTM standard D695-15^23^ for compression of additive manufactured components was followed. Ten samples were printed with five for the strength test and the other five for the modulus test. The speed of testing, which was 2 mm/min., and measurement procedures followed the specifications in^23^. The properties of the material are summarized in Table S2. The stress vs. strain data from the uniaxial compression test at room temperature was imported directly into ANSYS as the constitutive law; see a typical result in Fig. S3.

All the rods in Table S1 were modeled using ANSYS design modeler and analyzed under uniaxial compression. The same height (10 cm), boundary conditions, loading and meshing scheme were applied to all the rods for consistency. Static analysis and Euler’s buckling analysis were conducted to evaluate the rod response at a constant load. For buckling analysis, all the rods were modeled with the same uniaxial compression load (1000 N) with one end fixed and the other end pin supported. For each rod, ANSYS output a buckling factor, which when multiplied by the applied load (1000 N), gave the actual buckling load of the rod. A convergence analysis was conducted, and based on the results, the final element type was determined to be hexahedral and the element size was 0.1 mm. The buckling load, stress, displacement, mass and volume of all the rods were recorded from the model interface.

### Experimental validation

For the experimental validation, initially SolidWorks design tool was used to model some typical biomimetic rods. STL file formats were generated for the rods in order to be machine-readable. An extrusion-based 3D printer-Creality Cr-10 s at LSU was used to print these optimized rods using PLA filament. PLA with an extruder temperature of 210 °C and nozzle thickness of 1.75 mm was used. The 3D printer consists of three different resolutions from coarse to fine layer thickness. To compensate for the time constraint and maintain consistency, coarse resolution was employed for all the rods printed. Minimum post-processing was required for extrusion-based printing of these designs as only a few supports were required. The STL files of the drawings from SolidWorks were converted into a g-code using the Cura^24^ interface available online. These g-codes were fed into the printer to print the desired rods. Figure S4 shows several 3D printed biomimetic rods. Once all the rods were printed and cleared of the support material, a Q-TEST 150 testing machine with a capacity of 150KN was used for the compression testing. The test was conducted at 2 mm/min for all the rods. The mass of each rod was physically weighed prior to the tests. The buckling capacities, i.e., the load when the rods start to buckle were recorded.

### Feature identification or fingerprints

The first step in machine learning is to digitalize the biomimetic rods. Machine learning is artificial intelligence which can be used to train systems to learn from the data provided, which in turn can be used for predicting or categorizing new untrained data. It is very advantageous in reducing human intervention, complicated programming and computational time. The machine learning algorithm needs the rods to be fingerprinted. Fingerprinting refers to converting each individual rod into a machine-readable code or sequence. For this purpose, each different shape and feature of the designs are given a unique identification (number). For example, a bamboo-inspired column consists of an outer circular shape, inner circular surface and several smaller hollow cylinders of different diameters. These shapes differ from design to design. A unique number for the outer circle, inner circle and the rest of the smaller circles are assigned along with their location with respect to the origin of the coordinate system. It was made sure that the outer circle’s center lay on the origin for all the designs. For simplification, all the small circles that form porosity are designed to be of the same diameter. Similarly, each design with a new shape is given a unique number. Seven outer circles (1 to 7), six inner circles (8 to 13) and three smaller inner circles (14 to 16) are identified from the bamboo-inspired designs. These shapes are numbered and used to form the fingerprints. A similar procedure is followed for all the 1500 biomimetic rods in Table S1. A single vector consisting of different numbers including all the features in a rod was the final fingerprint for the rod. Some designs such as the root cross-section contain about 400 small spherical pores, which implies that the fingerprint vector comprises 400 variables plus the outer circle. For example, the fingerprint of a rod with outer, inner and ten small cylinders is of the form “1 (0,0), 9 (0,0), 14 (0.8, − 0.18333), 14 (0.71667, − 0.26667), 14 (0.56667, − 0.31667), 14 (0.76667, − 0.51667), 14 (0.56667, − 0.51667), 14 (0.36667, − 0.53333), 14 (0.45, − 0.71667), 14 (0.31667, − 0.65), 14 (0.11667, − 0.6), 14 (0.21667, − 0.75)”. It uniquely defines a rod that contains an outer circle (1 (0,0)), an inner circle (9 (0,0)) and then smaller circles (14 (0.8, − 0.18333), 14 (0.71667, − 0.26667), 14 (0.56667, − 0.31667), 14 (0.76667, − 0.51667), 14 (0.56667, − 0.51667), 14 (0.36667, − 0.53333), 14 (0.45, − 0.71667), 14 (0.31667, − 0.65), 14 (0.11667, − 0.6), 14 (0.21667, − 0.75)). The meaning of the numbers can be explained as follow. For example, for 14 (0.21667, − 0.75), the number 14 means it is a small circle or cylinder, and (0.21667, − 0.75) is the coordinate of the bottom center of the cylinder.

### Forward design and prediction

Machine learning can be formally perceived in two settings, supervised and unsupervised learning. Supervised learning implies data having known input and output values. On the contrary un-supervised learning consists of a set of inputs without labels. In this particular study, the data available (biomimetic rods with uniaxial buckling loads) have both the inputs, which are the structural properties such as mass, volume and microstructure, and the output, which is the buckling strength of individual structures (rods). Hence, supervised learning was used to train the dataset. All the rods were fingerprinted in the same manner as proposed above to maintain consistency. The mass, volume and buckling strength of all the designs were used directly as features. All the data were stored in an excel sheet containing the mass, volume, buckling strength and fingerprints of geometrical features of the individual rods. While approaching a supervised or un-supervised learning problem, many different algorithms are available and no one approach can be called superior to the others. Each type of problem and datasets have a suitable algorithm that works best. Supervised machine learning algorithms are being widely used in material and chemical engineering for property prediction and discovering new materials. Kernel Ridge Regression (KRR) has been used to handle non-linear relations in the property predictions of polymers^20^. Gaussian Process Regression (GPR) has been used by the same group identifying that it is more suitable for predicting a better uncertain/confidence interval of polymers and their properties^20^. Neural network models have been used by Stephen et al. to assist in the discovery of polymers with high thermal conductivity^25^. Support vector machines (SVM) which are considered to be very effective in real value function estimation have been used to predict the mechanical properties of cement^21,22^. Several other algorithms like decision trees, K-Nearest Neighbors, and gradient boosting algorithms have been proved to be effective in predicting properties with high accuracy^26^. Neural networks have been used to estimate the stress distribution in the aortic wall based on FEA results with an average discrepancy of 0.492%^27^. Regression tree has been used to predict the mechanical properties of carbon fibers like the longitudinal and transverse elastic modulus and shear modulus using data generated from finite element modeling^28^. Support Vector Regression models have been used to propose FEA models which can find a direct relationship between the input and output of the elements. This avoids the complex numerical iterations involved in finding the internal displacement field^29^. Ensemble methods have been used to model the bio-mechanical behavior of breast tissues under compression using results for FEA models^30^.

In this study, MATLAB was used to evaluate the dataset with different machine learning algorithms available. Ninety percent of the data were used for training the regression models and the remaining ten percent of the data were used for testing the performance of the various algorithms. The mass, volume and fingerprints were defined as the inputs and the buckling strength was defined as the output for all the regression models. Since the output is a single variable depending on multiple input variables that are correlated to each other, it is easy for the machine learning algorithm to formalize a relationship between them. Fivefold cross-validation was used to evaluate the performance of the machine learning algorithms in predicting new data. MATLAB allows us to check the performance of various pre-programmed machine learning algorithms with our data and gives a root mean square error (RMSE) of each model prediction. It is the difference between the predictions and actual values of the observations. It was observed that the Ensemble Bagged Tree algorithm with a leaf size of eight is the best-suited machine learning algorithm based on the RMSE as compared to other models like SVM, GPR and neural networks for the data type being analyzed. In this model, an ensemble tree divides the dataset into different subsets and trains each subset individually. The average of all the subset model predictions was used as the final prediction^31^. The ensemble of all the subsets gave a much robust model as compared to individual subsets.

### Optimization

The further step in designing biomimetic rods is to optimize these designs to develop even better rods with superior buckling strengths. The advantage of using a machine learning program for optimization is the exceptional speed in validating new designs and also ease in designing. The program needs to be fed with just the fingerprints of the new designs to predict their buckling loads. For the optimization, the developed machine learning algorithm is used for forward prediction of various untrained fingerprints. It is fairly easy to develop a code that generates different fingerprint patterns compared to manually designing each structure individually. Therefore, initially a MATLAB code is programmed to generate all the possible combinations inspired from the biomimetic rods. This generates more than a million combinations. However, not all combinations exhibit finer structural properties. From the first few datasets, a clear pattern (number of internal micro structures) can be manually observed which helped in defining a minimum and maximum porosity for the rods to perform better compared to the semi optimal (1,500 biomimetic) rods. This initially helped in truncating the data sets manually. For further filtering of non-optimal designs, EXCEL and MATLAB functions are (can be) used. In EXCEL^32^, “IF” function and “>” or “<” can be used to identify the numbers greater or less than a given value (buckling load in this case) and the “Index” function can be used to display the filtered fingerprints. MATLAB does not have a function for indexing. But the “>” or “<” can be used to identify the desired fingerprints from the predicted dataset and the identified variables (fingerprints) can be called and defined into a new dataset. Finally, a dataset that contained 160 new designs (fingerprints) which exhibited better buckling properties compared to the biomimetic rods in the training dataset was generated. The optimum designs which have higher buckling strengths were taken to evaluate their performance. These new fingerprints were converted into 3D CAD designs using ANSYS and analyzed under uniaxial compression for their structural properties.

## Supplementary Information

Supplementary Information 1.

Supplementary Information 2.

## Data Availability

All other data are available from the authors upon reasonable request.
