# Supplementary material for: 3D printable biomimetic rod with superior buckling resistance designed by machine learning
Source: Sci Rep. 2020 Nov 26;10:20716. doi: 10.1038/s41598-020-77935-w (PMC7692558; doi:10.1038/s41598-020-77935-w)
Supplement: Supplementary file 1 — Supplementary Information 1. [file 41598_2020_77935_MOESM1_ESM.docx]

Supplementary Information for

**3D printable biomimetic rod with superior buckling resistance**

**designed by machine learning**

Adithya Challapalli, Guoqiang Li

This file includes:

Figures S1-S4

Tables S1-S5


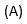


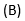


**Fig. S1. (A) Buckling analysis and (B) stress analysis of a few of the rods designed from biomimetic structures** (t1-t13 represent a few of the biomimetic rods; see **Table S4** for details).


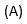


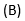


**Fig. S2. (A) Compressive stress and (B) axial displacement of optimized rods with respect to mass.**

**Fig. S3. Stress vs strain curve for a typical 3D printed compression test sample**

**
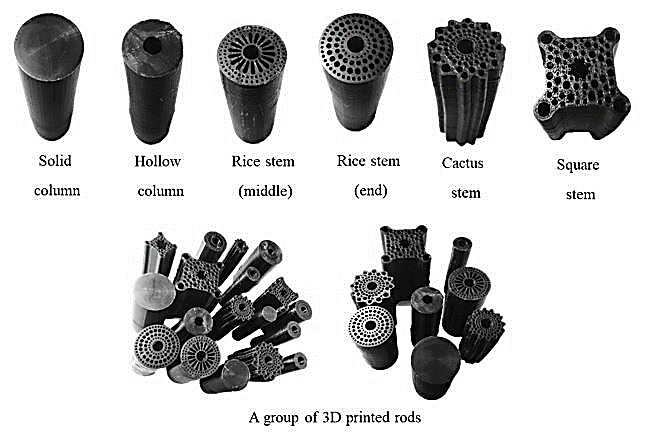
**

**Fig. S4. 3D printed biomimetic rods.**

**Table S1. The created biomimetic rods for finite element analysis and training dataset**

| Biomimetic inspiration for external shape | Primary designs for internal microstructure (a total of 21) | Extended dataset by adjusting the internal structures (a total of 1,500) |
| --- | --- | --- |
| Bamboo, rice | 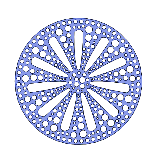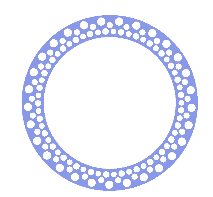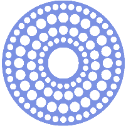 | 91 dummy rods |
| Square plant | 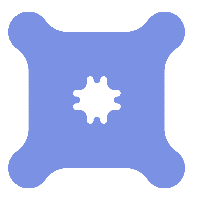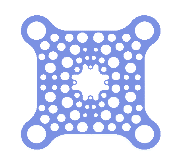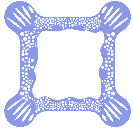 | 81 dummy rods |
| Bulrush | 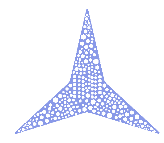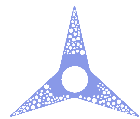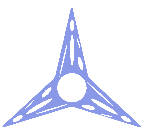 | 71 dummy rods |
| Cactus | 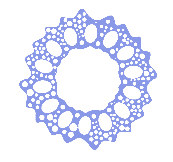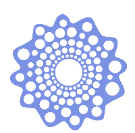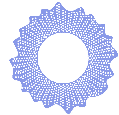 | 81 dummy rods |
| She-oak | 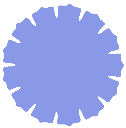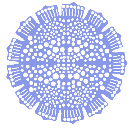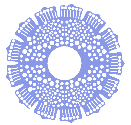 | 50 dummy rods |
| Sedge | 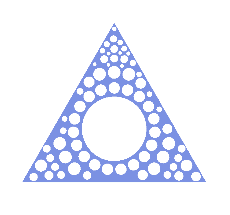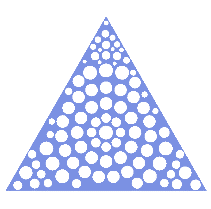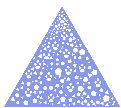 | 50 dummy rods |

**Table S2. Mechanical properties of 3D printable PLA**

| Material | PLA |
| --- | --- |
| Density | 1,138 kg/m^3^ |
| Poisson’s ratio | 0.4 |
| Young’s modulus | 1.2424 $\mathrm{GPa}$ |
| Compressive strength | 55.3 $\mathrm{MPa}$ |
| Tensile strength | 11 $\mathrm{MPa}$ |

**Table S3. Buckling mode comparisons of different rods (A) FEA and (B) experiments**

| Name | 1. Deformed shape by ANSYS | 1. Deformed shape by experiment |
| --- | --- | --- |
| Solid column | 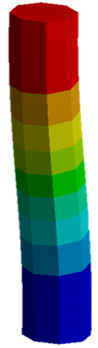 | 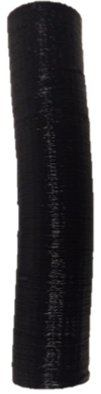 |
| Bamboo stem | 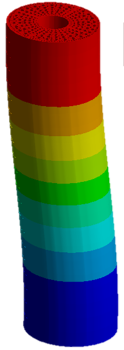 | 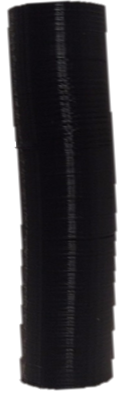 |
| Cactus stem | 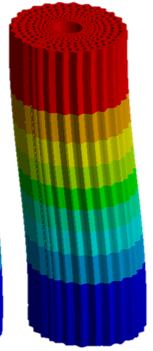 | 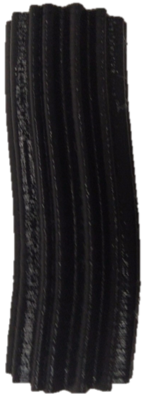 |

**Table S4. Different biomimetic rods represented in Figure S1**

| t1 | Hedgehog quill cross-section | 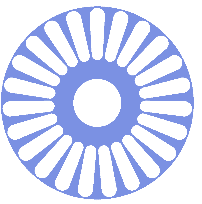 |
| --- | --- | --- |
| t2 | Cactus stem dummy rod | 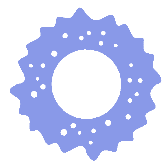 |
| t3 | Square stem | 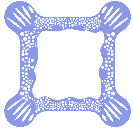 |
| t4 | Banana stem | 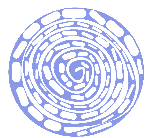 |
| t5 | She-oak stem | 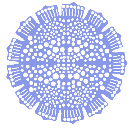 |
| t6 | Sedge stem | 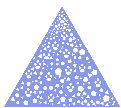 |
| t7 | Square stem dummy rod | 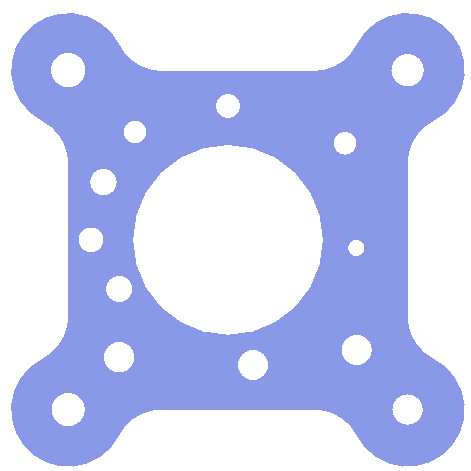 |
| t8 | Clematis (Hexagonal) stem | 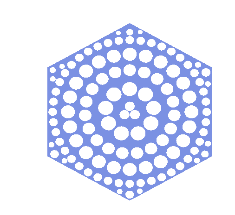 |
| t9 | Nymphaea stem | 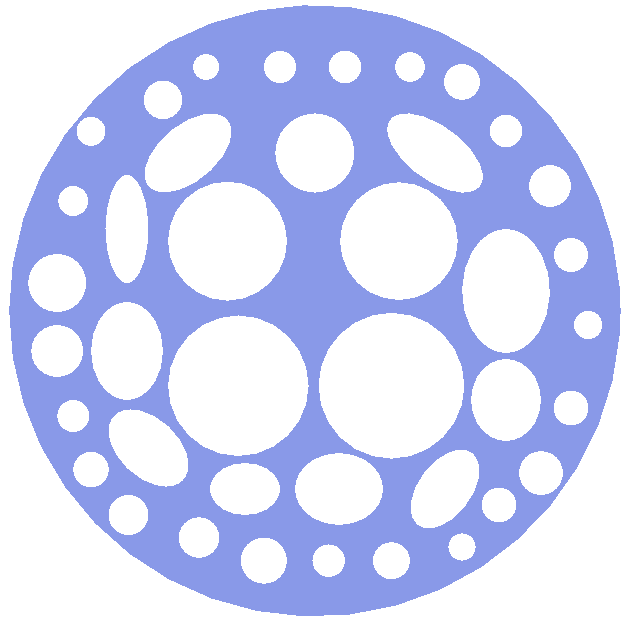 |
| t10 | Sea-shell | 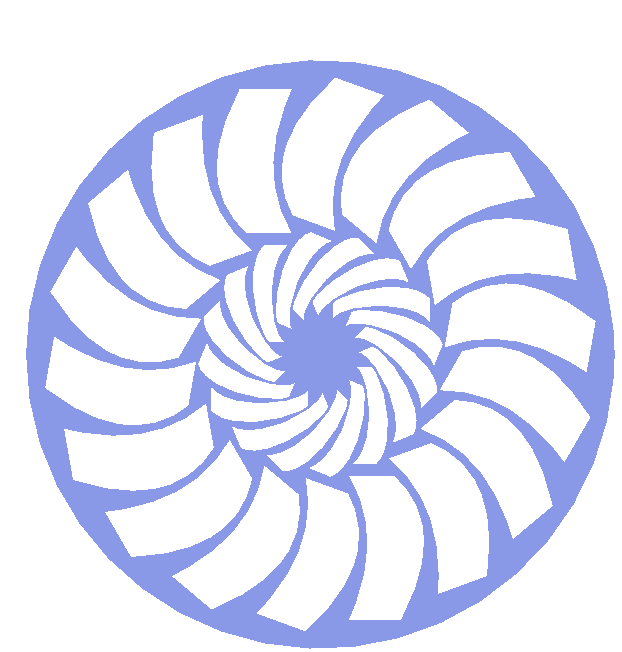 |
| t11 | Hollow cylinder with groves  (Inspired from sea shells) | 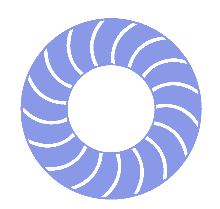 |
| t12 | Bamboo stem | 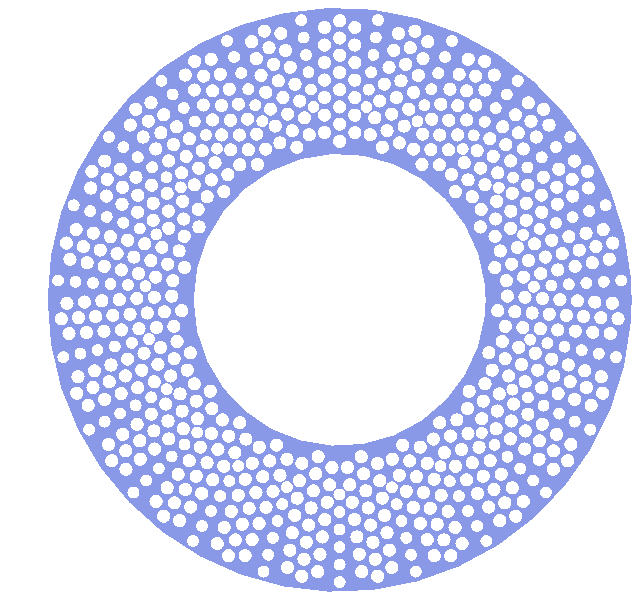 |
| t13 | Bamboo stem dummy rod | 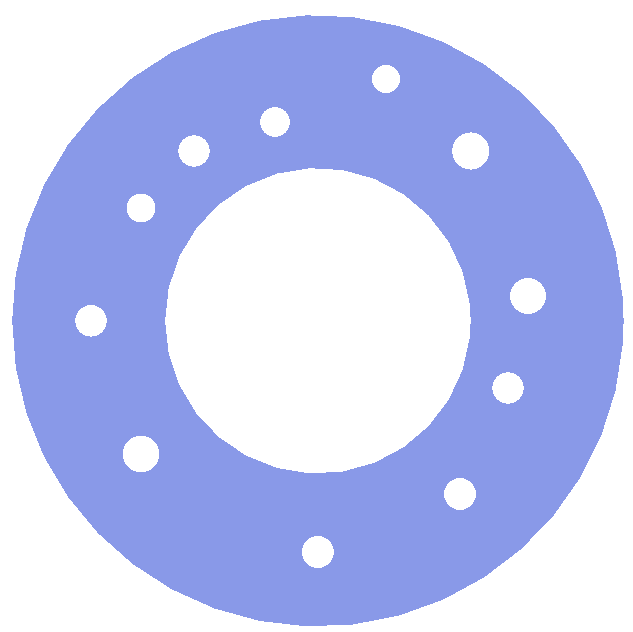 |

**Table S5. The created optimal designs from inverse design and optimization**

| External shapes of optimized biomimetic designs | Primary designs with optimized internal shapes | Number of optimal designs in each type |
| --- | --- | --- |
| Bamboo | 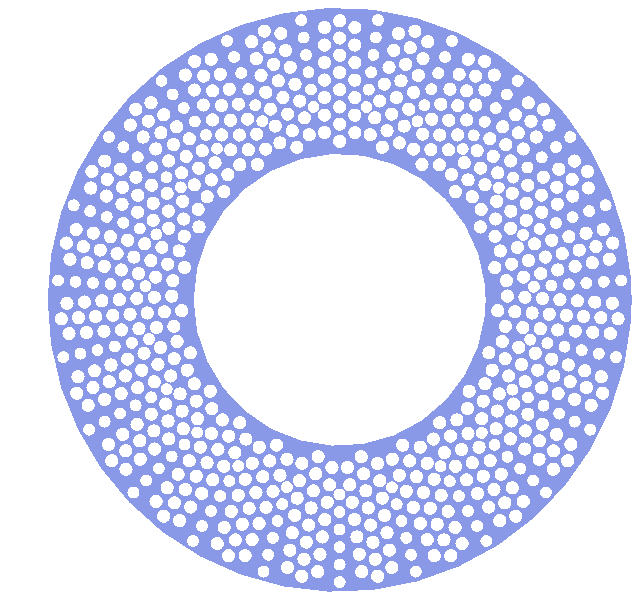 | 50 rods |
| Square plant | 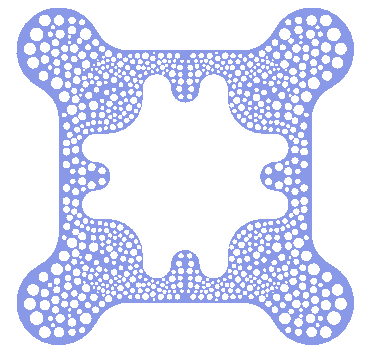 | 40 rods |
| Cactus | 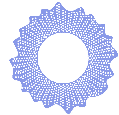 | 50 rods |
| Bulrush | 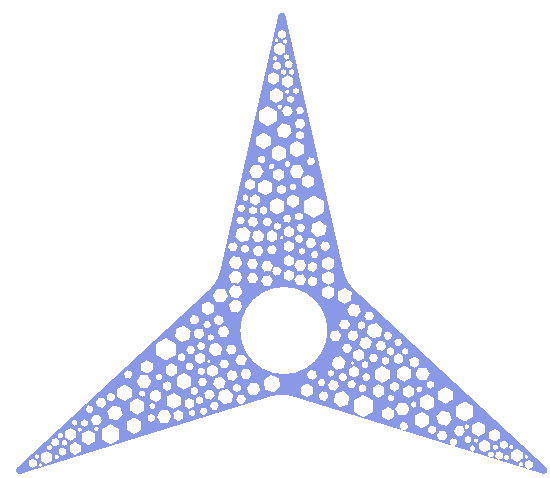 | 20 rods |
